# Optimizing Crop Planting Schedule Considering Planting Window and Storage Capacity

**DOI:** 10.3389/fpls.2022.762446

**Published:** 2022-03-02

**Authors:** Saiara Samira Sajid, Guiping Hu

**Affiliations:** ^1^Department of Industrial and Manufacturing Systems Engineering, Iowa State University, Ames, IA, United States; ^2^Rochester Institute of Technology, Rochester, NY, United States

**Keywords:** mixed-integer linear programming, time series data, 1D-convolutional neural networks, TBATS, storage capacity, planting window

## Abstract

Technology advancement has contributed significantly to productivity improvement in the agricultural sector. However, field operation and farm resource utilization remain a challenge. For major row crops, designing an optimal crop planting strategy is crucial since the planting dates are contingent upon weather conditions and storage capacity. This manuscript proposes a two-stage decision support system to optimize planting decisions, considering weather uncertainties and resource constraints. The first stage involves creating a weather prediction model for Growing Degree Units (GDUs). In the second stage, the GDUs prediction from the first stage is incorporated to formulate an optimization model for the planting schedule. The efficacy of the proposed model is demonstrated through a case study based on Syngenta Crop Challenge (2021). It has been shown that the 1D-CNN model outperforms other prediction models with an RRMSE of 7 to 8% for two different locations. The decision-making model in the second stage provides an optimal planting schedule such that weekly harvested quantities will be evenly allocated utilizing a minimum number of harvesting weeks. We analyzed the model performance for two scenarios: fixed and flexible storage capacity at multiple geographic locations. Results suggest that the proposed model can provide an optimized planting schedule considering planting window and storage capacity. The model has also demonstrated its robustness under multiple scenarios.

## Introduction

With the current world population growth rate, it is anticipated that by the end of 2050, the agriculture system needs to support 10 billion people, and 3 billion increase from the current population (Ranganathan et al., 2018). To address this challenge, innovations in agricultural production are necessary to feed the increasing population. It should be noted that significant advancement has been made in crop breeding to improve the yields. However, the inefficiency in crop logistic management during harvesting and storage can constitute 30–50% of produced food wasted yearly, which is around 2 billion metric tons (Fox, 2013). Motivated by this challenge, we designed a planning tool to improve the management during crop planting, harvesting, and storage. The proposed tool provides a scheduling model for crop planting, providing consistent weekly harvest quantity. The algorithm designed for the crop planting ensures that an optimum planting schedule is designed that will have even weekly harvested quantities within storage capacity and the planting dates are within preferred planting timeframe. It should be noted that it is much more convenient to map out the storage of evenly spread harvested quantities compared to erratic quantities; therefore, this tool is expected to contribute to the decision-making of breeding companies and farmers. On a broader view, the planning tool will mitigate the operational and productivity challenges, subsequently reduce yield loss from the harvesting and storage phases.

Lauer et al. (1999) claimed that crop breeds or populations tend to have higher yields when planted within a specific time window, and ignoring the planting window will reduce yield (Swanson and Wilhelm, 1996; Darby and Lauer, 2002; Anapalli et al., 2005; Williams, 2006; van Roekel and Coulter, 2011). In addition, interactions between the planting date and soil temperature (Bollero et al., 1996) and interactions between the planting date and fertilizer application (Hankinson et al., 2015; Kaiser et al., 2016) have a significant impact on crop yield irrespective of geographical location (Beiragi, 2011; Tsimba et al., 2013). Therefore, it is of paramount importance to stay in the preferred planting window. Our proposed scheduling model for crop planting ensures that planting is performed within the recommended planting window.

Crop production and operation can be divided into multiple stages: field preparation, planting, fertilizer application, growing, harvesting, and storage (Polgár et al., 2020). To achieve an optimal planting schedule that will bring about a consistent weekly harvesting quantity, it is vital to determine these stages correctly. Plant development is contingent upon specific heat requirements, soil moisture content, and geographical location (Sacks et al., 2010). For a particular geographic area, these growth stages can be measured by accessing growing degree units (GDUs) or days (GDDs), which is a numeric value calculated based on daily heat value (Bonhomme, 2000; Miller et al., 2001). Briefly, GDUs are measured in terms of Celsius or Fahrenheit for each day, whereas GDD will be zero if the average temperature of a day is less than a base temperature else it will be one. Even though the calculations for GDUs and GDDs are different they provide similar weather information. After planting, crops grow and become ready for harvesting once the total heat absorption reaches a specific value. As these heat requirements are measured in terms of GDUs or GDDs, plants are considered ready for harvesting after reaching required GDUs or GDDs. Two methods have been designed and adapted to calculate GDUs or GDDs, temperature averaging and the Baskerville-Emin method (Battel, 2021). Since GDUs give an estimation of the development stage, an accurate prediction of the GDUs is essential to identifying when plants will be ready for harvesting. When the accumulated GDUs reach the required threshold, the plants are considered to be ready for harvesting, and the week is defined as harvesting week. Harvesting with significant deviation from crop maturity would impact crop yield. Subaedah et al. (2021) suggested that crops harvested before reaching required GDUs will reduce crop yield. Similarly, Thomison et al. (2011) found in their research that delaying harvesting beyond crop maturity will reduce the yield. By accurately predicting GDUs, crop maturity dates can be evaluated more accurately. Therefore, an accurate GDUs prediction model becomes essential to predicting the harvesting week.

Various techniques have been studied to predict GDUs, such as the linear regression model (Neild and Seeley, 1977), the non-linear model (Zhou and Wang, 2018). Wypych et al. (2017) found that GDUs are sensitive to climate change. However, most existing methods did not consider the impact of climate change in GDUs prediction. To include the effect of climate change in GDUs, we predicted GDUs through time series analysis of daily historical data. For time series analysis and prediction, TBATS, which stands for Trigonometric Exponential Smoothing with Box-Cox transformation, ARMA errors, Trend, and Seasonal decomposition, has been widely adopted in research (Shin and Yoon, 2016; Cherrie et al., 2018; Naim et al., 2018; Gos et al., 2020; Gonçalves et al., 2021). In our study, TBATS has been chosen as a benchmark prediction model. In addition, convolutional neural networks (CNN) have been applied to predict time series data (Oh et al., 2018). It should be noted that designing the 1D-CNN architecture with appropriate kernel size, the number of layers and filter size by parameter tuning plays a vital role in prediction (Tang et al., 2020). Therefore, we proposed a 1D-CNN with optimized parameters to improve the prediction accuracy. Inspired by the performance of TBATS and 1D-CNN in time series analysis, we applied them for the prediction of GDUs.

In the agricultural sector, various models are proposed to determine optimal yield quantity (Nafziger, 1994; De Bruin and Pedersen, 2008; Hörbe et al., 2013; Waongo et al., 2015). However, limited research focusing on planting schedules has been identified in the existing body of literature. Swaminathan and Zhang (2021) proposed an optimal planting schedule to improve crop yield considering planting capacity, soil water content, etc. A randomized blocked design experiment was conducted to determine the impact of different planting and harvesting periods on the growth of the Acorus calamus (Gyalpo Bhutia and Bhutia, 2018). Linear optimization models have been used to develop optimal planting schedules for different crops (Indriyanti et al., 2019; Wang et al., 2020). The utilization of linear optimization models to build planting models has motivated us to further exploration of this field.

Customized crop models are required depending upon the constraints as one model may not address all requirements (Boote et al., 1996). To the best of our knowledge, little research has considered both planting window and storage capacity while proposing an optimal planting schedule. This research focuses on this gap. A planting schedule that can lead to consistent weekly harvesting quantities will aid in reducing food wastage in the harvesting phase. Thus, we propose a scheduling model for planting, considering both the planting window and storage capacity while providing optimal planting dates. The focal research question in this study is to design an optimal planting schedule that will lead to consistent weekly harvesting quantity within storage capacity in the harvesting phase. This problem statement is based on the INFORMS Syngenta Crop Challenge 2021 (Syngenta Crop Challenge (2021) – the challenge). We developed a mixed-integer linear programming (MILP) crop planting model; the objective is to ensure a consistent weekly harvest quantity with minimum harvesting weeks while maintaining the planting window and storage capacity. Furthermore, a user interface combined with the proposed model will help users dynamically update planting strategies (Johnson, 2013; Athirah et al., 2020).

The rest of the manuscript is organized as follows. In section “Materials and Methods,” the proposed methods are discussed in detail, along with the modeling framework and solution algorithm. In section “Case Study,” a case study based on a real-world application has been presented to explain and illustrate the proposed prediction and decision-making framework. Finally, the research findings and conclusions based on this study are discussed in section “Conclusion.”

## Materials and Methods

The proposed plant scheduling model recommends an optimal planting schedule considering the storage capacity after harvesting. This model comprises two vital stages, the first stage focuses on determining the plant maturity date for harvesting, and the second stage delivers the planting schedule. Since GDUs provide an estimate of plant growth, in the first stage GDUs are predicted to specify harvesting date. These obtained harvesting dates are one of the inputs to the subsequent stage to attain the planting schedule. In the second stage, storage capacity is considered while providing the planting schedule. Particularly, the research problem statement along with modeling assumptions are based on the INFORMS Syngenta Crop Challenge 2021 specifications (Syngenta Crop Challenge, 2021 – the challenge).

**FIGURE 1:**
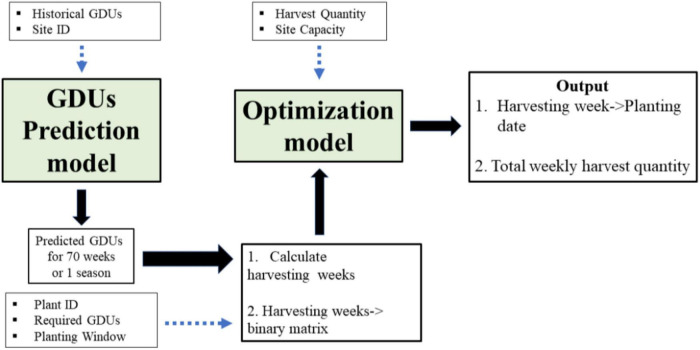
Schematic diagram of the planning process.

The entire process of the proposed model is presented by a schematic diagram in Figure 1, where the two model stages are in green boxes, and the blue dotted lines show the input to those stages from field data. The GDUs prediction model has two inputs: location or site ID and historical GDUs. In addition to the harvest quantity of crop breeds and storage capacity, the predicted GDUS are input to the optimization model. The final output of the process is the harvesting week for each population, along with the weekly harvesting quantity. The planting date for each population is calculated from the harvesting week through a transformation function, which will provide the optimal planting schedule for a combination of a specific location and a set of crop breeds or populations.

### Growing Degree Units Prediction

Accurate weather and climate prediction are essential in farm management to reduce risk and make optimal decisions (Templeton et al., 2014). In this analysis, we have performed GDUs predictions, incorporating historical weather data, to make decisions about harvesting week. Prediction of GDUs involves a time series analysis of daily data to estimate GDUs for the upcoming season. Depending on crop type, the season length may vary, which requires the prediction model to be capable of predicting GDUs for an extended period with better accuracy. The time-series data may have multiple seasonality within a cycle, which poses additional challenges for the prediction model. For the time series analysis, one well-established method, TBATS, is compared with a comparatively new method, 1D-CNN. For demonstration purposes, both approaches are applied in two locations, named site 0 and site 1. Comparing the results of both models, we selected the 1D-CNN model with higher prediction accuracy. The efficacy of 1D-CNN suggests that it can be applied to other geographic locations for GDUs prediction. In the following sections, a brief description of the TBATS model and 1D-CNN model is provided.

#### TBATS

The governing equations for the TBATS model are given by Eqs a–i, which was proposed by de Livera et al. (2011). A detailed explanation for the model parameters can be found in the manuscript. This model requires initial parameters or seeds to start the prediction, where the number of these parameters depends on the number of seasonality denoted by *k*. The modeling parameters are *l_0_*, *b_0_*, $\left\{s_{0}^{1},s_{0}^{2},\ldots \,s_{0}^{m_{1}}\right\}$, {$s_{0}^{1},s_{0}^{2},\ldots s_{0}^{m_{2}}\}$, {$s_{0}^{1},s_{0}^{2},\ldots s_{0}^{m_{k}}\}.$The smoothing parameters include α,β,γ_1_,γ_2_….. γ_*k*_, and ω is the parameter for Box-Cox transformation. The trigonometric components, given by Eqs h, i, address the stochastic level of seasonal components. The parameter, ε_*t*_, is a Gaussian error factor.


(a) $$y_{t}^{\left(\omega \right)}=\left\{ \begin{array}{c} \frac{y_{t}^{\omega }-1}{\omega },\omega \neq 0\\ \log y_{t},\omega =0 \end{array}\right.$$

(b) $$y_{t}^{\left(\omega \right)}=l_{t-1}+\emptyset \,b_{t-1}+\sum_{i=1}^{T}s_{t-m_{i}}^{\left(i\right)}+d_{t}$$

(c) $$l_{t}=l_{t-1}+\emptyset \,b_{t-1}+\alpha \,d_{t}$$

(d) $$b_{t}=\left(1-\emptyset \right)\,b+\emptyset \,b_{t-1}+\beta \,d_{t}$$

(e) $$s_{t}^{\left(i\right)}=s_{t-m_{i}}^{\left(i\right)}+\gamma_{i}\,d_{i}$$

(f) $$d_{t}=\sum_{i=1}^{p}\varphi_{i}\,d_{t-i}+\sum_{i=1}^{q}\theta_{i}\,\varepsilon_{t-i}+\varepsilon_{t}$$

(g) $$s_{t}^{\left(i\right)}=\sum_{j=1}^{k_{i}}s_{j,t}^{\left(i\right)}$$

(h) sj,t(i)=sj,t-1(i)⁢cos⁡λj(i)+sj,t-1(i)*⁢sin⁡λj(i)+γ1(i)⁢dt

(i) sj,t(i)*=-sj,t-1(i)⁢sin⁡λj(i)+sj,t-1(i)*⁢cos⁡λj(i)+γ2(i)⁢dt

$$\varepsilon_{t}∼N\,I\,D\,\left(0,\sigma^{2}\right)$$

**FIGURE 2:**
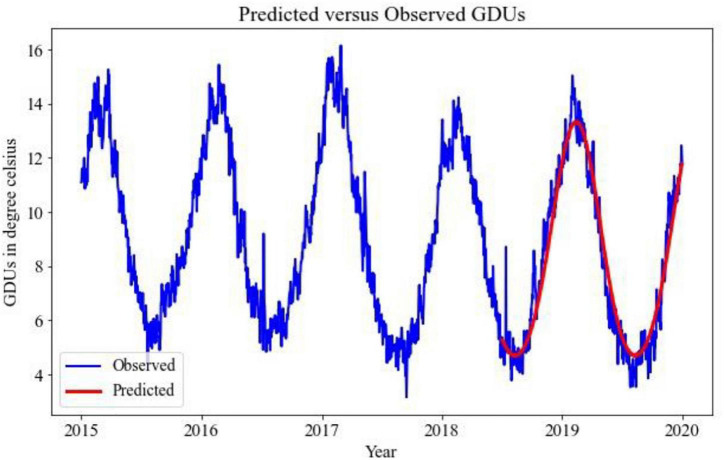
TBATS GDUs prediction for site 0 for the year 2018–2019.

**FIGURE 3:**
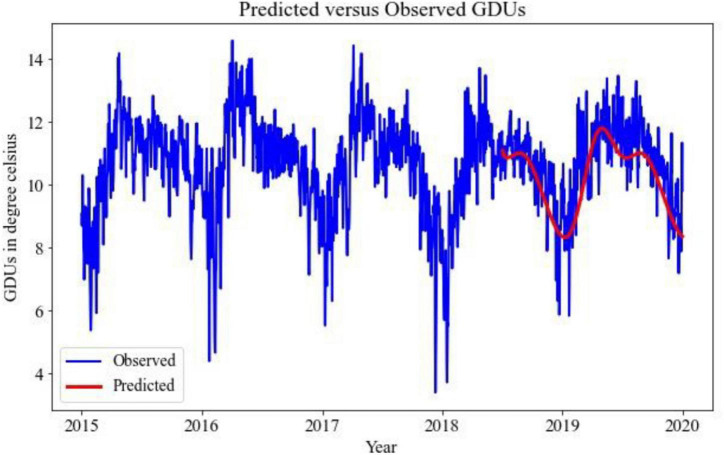
TBATS GDUs prediction for site 1 for the year 2018–2019.

Implementing the TBATS model for the prediction of GDUs for two different locations, the results are shown in Figures 2, 3, where the blue lines are the observed GDUs, and the red lines are the predicted values. All the parameters used for TBATS model are the default parameters specified by TBATS package in Python except the cycle length. While predicting GDUs, the length of a single season, k is considered one year or 365.25 days to address the leap year. It is observed that for site 0, in Figure 2, GDUs vary in the range of 4–16^°^C with comparatively less variance than site 1.

In Figure 3, for site 1, GDUs have a similar range to site 0 but with a higher variance. Another observation for site 1 is, GDUs are mainly above 10^°^C, whereas, for site 0, mostly GDUs are below 10^°^C. Because, variance in site 1, it has a higher RRMSE than for site 0. Therefore, one observation is TBATS model has better accuracy for data with limited variance.

#### One-Dimensional Convolutional Neural Networks

The second method to predict GDUs is based on neural networks. For time-series data, among several neural models, two types of neural network models have been widely adopted in the literature. One is recurrent neural networks (RNN), and the other is one-dimensional convolutional neural networks (1D-CNN). 1D-CNN has an advantage over RNN since it requires fewer parameters to tune (Zhao et al., 2020). Therefore, we adopted 1D-CNN for prediction in this study. The first step is to preprocess the time-series data to a data frame with features and response variables. Appropriate network architecture is then selected after preprocessing the data to a required form.

##### Data Pre-processing

**FIGURE 4:**
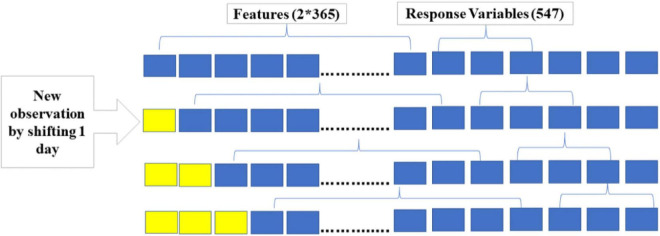
Data preprocessing for 1D-CNN.

One of the challenges in utilizing CNN to predict time series data is to create features and response variables. The first step is to specify the number of days to be considered for prediction, which will be the number of features. The second step is to define the length of the prediction period, and this will give the number of response variables the model needs to predict. The 1D-CNN model developed for this analysis considers GDUs of the past 2 years to predict GDUs of the next 1.5 years. Meaning that on the nth day, using GDUs of n-2*365 days, GDUs for the next 547 days or 1.5 years from day n will be predicted. This process is illustrated in Figure 4, the first observation is the first 2*365 days as features and the next 547 days are the response variables. The second observation is created by shifting the 2*365 days window by one day and selecting the next 547 days as response variables. This process is repeated until all data have been preprocessed. The training set includes 2011 to the first half of 2019, and the test set is from the second half of 2019 to 2020. The model is trained on the dataset of the first 8.5 years. Once the model is trained, it can predict GDUs for the next 1.5 years or 547 days, given the GDUs of the past 2 years. This process can be utilized for any season length by modifying the length of the prediction period, meaning increasing or decreasing the number of response variables.

##### Model Architecture

**FIGURE 5:**
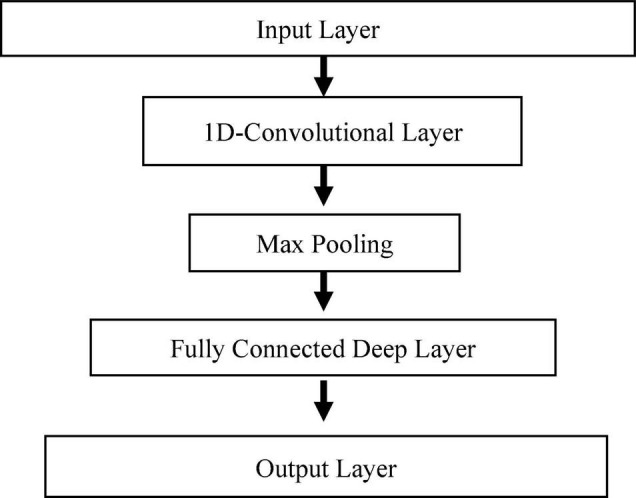
Proposed 1D CNN architecture.

The 1D-CNN architecture used for the prediction model is presented in Figure 5. After analysis of several architectures, a four-layer 1D-CNN is developed for the prediction model. The first layer is the input layer, which is followed by a convolution layer. After the convolutional layer, a max-pooling layer is added to reduce the size, and then a fully connected layer is added with 100 nodes. The last layer is the output layer, which gives predicted GDUs for the next 547 days. The number of nodes in the output layer can be modified based on the season length of the crop. A detailed description of the parameters used for the model is shown in Table 1. These parameters were selected based on trial and error and the best performing model was selected for prediction.

**TABLE 1:** 1D-CNN hyper-parameters.

Input layer	Shape : (1, 365*2, 1)
Convolution layer	Filters: 128 Kernel size: 2 Activation function: Relu
Max-pooling	Pool size = 2
Fully connected layer	No. of nodes = 100 Activation function: Relu
Output layer	No. of nodes = 547

**FIGURE 6:**
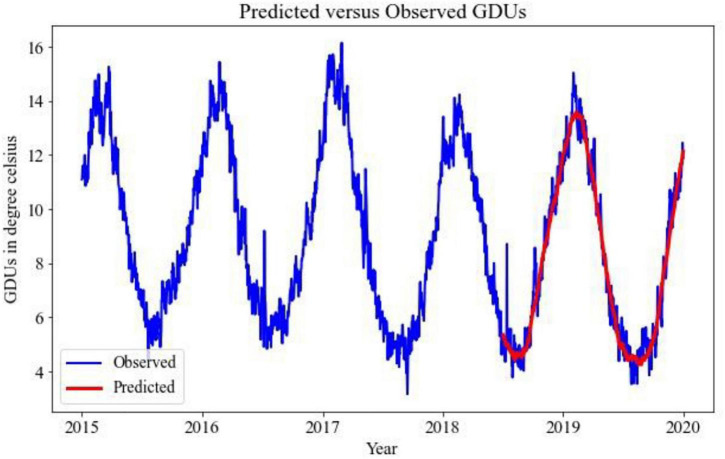
1D-CNN GDUs prediction for site 0 for the year 2018–2019.

**FIGURE 7:**
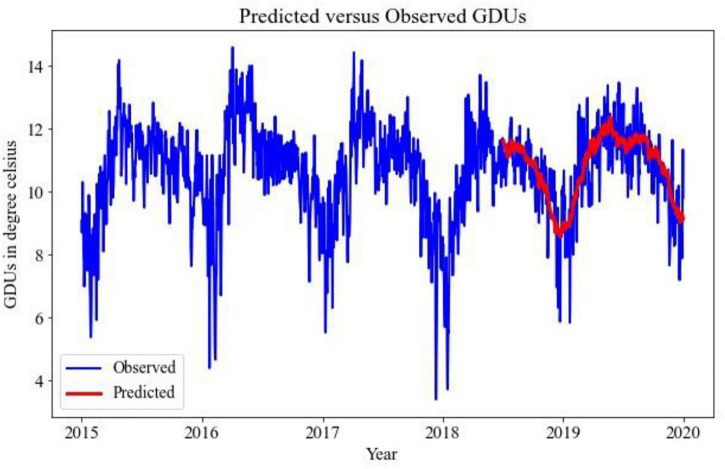
1D-CNN GDUs prediction for site 1 for the year 2018–2019.

The predicted results using 1D-CNN are included in Figures 6, 7 for site 0 and site 1, respectively. The 1D-CNN model is capable of making a good balance between bias and variance. Comparing to the TBATS model, the 1D-CNN model can better address the variance in data. In contrast, TBATS tends to smooth the prediction because of the Box-cox transformation and the trigonometric components.

A comparative analysis between TBATS and 1D- CNN is given in Table 2. It is evident that the 1D-CNN model outperforms benchmark TBATS for both locations in terms of RRMSE. Both in site 0 and site 1, 1D-CNN has RRMSEs of 7 and 8%, respectively, which are less than the RRMSEs of TBATS models. TBATS model has some limitations for long-term prediction, which may cause TBATS to have comparatively poor performance for predicting GDUs for the next 547 days. As a result, the 1D-CNN model has been selected to predict GDUs, and the outputs are utilized in the following steps.

**TABLE 2:** Comparative analysis of TBATS and 1D CNN results.

Site	Site 0		Site 1	
Model	MSE	RRMSE (%)	MSE	RRMSE (%)
TBATS	0.49	9	0.97	10
1D-CNN	0.30	7	0.65	8

### Optimization Model for Planting

The second stage is to formulate an optimization model for the planting decisions under varying weather conditions and locations for multiple population types. In this stage, the predicted GDUs from stage one is given as the input to the planting model for respective locations. The performance of the planting model is susceptible to the predicted GDUs. Therefore, it is essential to ensure the accuracy of the predicted GDUs. The planting model is developed based upon the following assumptions:

1. The harvested quantities for each crop breeds or population for respective scenarios are considered known.
2. Different scenarios and locations are considered mutually exclusive, which means storage capacity cannot be fixed and flexible simultaneously.
3. One population can only be harvested in 1 week.
4. A maximum value for the days required for harvesting is set, depending on the crop types.

The model formulation is for two different cases; in case-1, fixed-storage capacity is considered, only optimal planting date is to be identified. Whereas, in the second case, no fixed storage capacity is considered. For case-2, both optimal storage capacity and planting date are determined. Case-2 is applicable when storage capacity is flexible and has to be determined before the planting phase.

The model parameters used to build optimization model are listed as follows with a brief description of each model parameter.

Parameters:


$$P_{i\,E}=\mathrm{Earliest}\,\mathrm{planting}\,\mathrm{date}\,\mathrm{of}\,\mathrm{population}\,\mathrm{type}\,i$$

$$P_{i\,L}=\mathrm{Latest}\,\mathrm{planting}\,\mathrm{date}\,\mathrm{of}\,\mathrm{population}\,\mathrm{type}\,i$$

$$P_{i\,d}=\left\{ \begin{array}{c} d,\mathrm{if}\,\mathrm{population}\,i\,\mathrm{can}\,\mathrm{be}\,\mathrm{planted}\,\mathrm{on}\,\mathrm{day}\,d,\forall d\in \left[P_{i\,E},P_{i\,L}\right]\\ 0,\mathrm{else} \end{array}\right.$$

$$C_{s}=\mathrm{Given}\,\mathrm{site}\,\mathrm{capacity}\,\mathrm{for}\,\mathrm{site}\,s,s\in \left\{0,1\right\}$$

Qic=Harvest⁢quantity⁢of⁢population⁢type⁢i⁢for⁢scenario⁢c,c∈{1,2},i⁢n⁢e⁢a⁢r⁢s⁢c⁢o⁢r⁢n⁢c⁢o⁢u⁢n⁢t

$$H_{i\,d}=\left\{ \begin{array}{c} \mathrm{Week}\,\mathrm{number}\,\left(P_{i\,d}\,\mathrm{Days}\,\mathrm{requied}\,\mathrm{to}\,\mathrm{achieve}\,\mathrm{required}\,\mathrm{GDU}\,s\right)\\ 0,\text{else} \end{array}\right.$$

$$a_{i\,j}=\left\{ \begin{array}{c} 1,\mathrm{if}\,\mathrm{population}\,i\,\mathrm{is}\,\mathrm{harvested}\,\mathrm{in}\,\mathrm{week}\,j,H_{i\,d}=j,\\ \forall j\in \left\{1,2,3,\ldots \,\ldots \,70\right\}\\ 0,\text{else} \end{array}\right.$$

*P*_*iE*_ and *P*_*iL*_ are the earliest and the latest planting date of the planting window for plant *i*. *P*_*id*_, transforms the planting window into a matrix form, where *P*_*id*_ is equal to day of year(*d*) when the day is within planting window of plant *i*. *C_s_* is the storage capacity if site *s* and Qic is the harvest quantity of population *i* for scenario *c.* The formulated model ensures that the proposed planting dates are within the recommended planting window for different crop breeds or populations. However, to do so, planting window is not included as a direct input; instead, it considers the harvesting window as a constraint. This harvesting window is calculated from the planting window and predicted GDUs. For any crop breed, harvesting week corresponding to planting date within the planting window is calculated by, *H*_*id*_. A binary matrix *a*_*ij*_, is evaluated, which can have a value of 1, if particular crop breed *i*, can be harvested in week *j*. The pseudocode to generate *a*_*ij*_, is given in Table 3.

**TABLE 3:** Pseudocode to generate the binary matrix a_*ij*_.

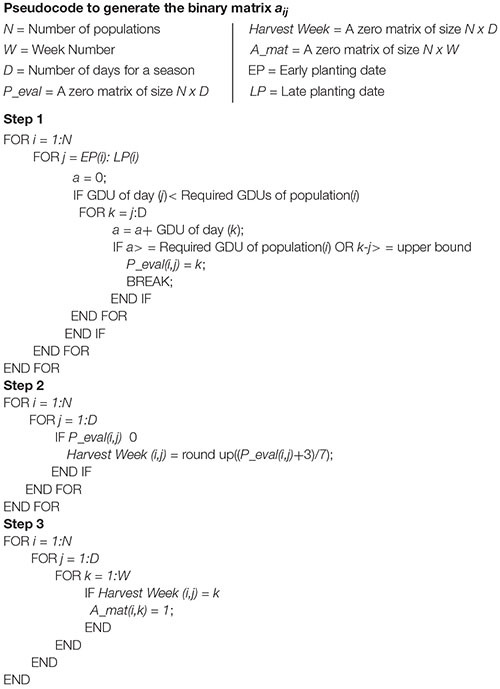

The pseudocode is divided into three steps. In the first step, for each crop breed, the harvesting date is calculated corresponding to each day in its recommended planting window. The days are calculated based on required GDUs for the respective crop breed, and also an upper limit is set for the required days of harvesting. In the second step, harvesting weeks corresponding to those harvesting days are calculated. In the last step, *a*_*ij*_, is created by putting 1 to harvesting week *j*, for which particular crop breed *i*, can be harvested. This *a*_*ij*_,is used in the model to put an upper bound on the binary decision variable *X*_*ij*_, which will ensure that any crop breed can only be harvested on weeks that respect their planting window.

#### Case-1: Fixed Storage Capacity

**FIGURE 8:**
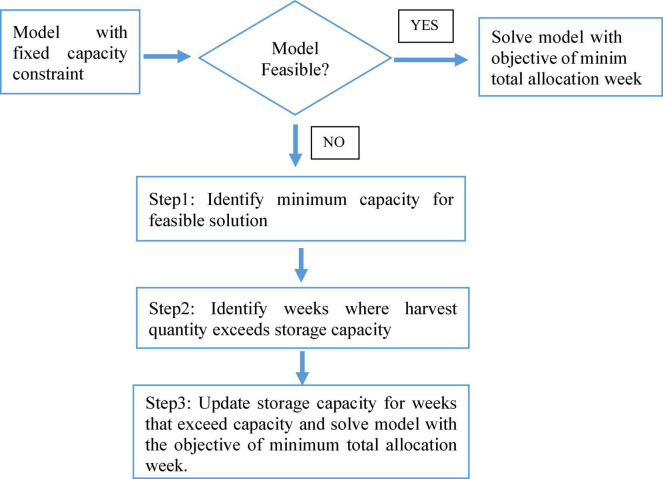
Proposed algorithm for optimal planting schedule with the fixed-storage capacity.

An algorithm has been developed to generate an optimal planting schedule for a fixed-storage capacity, as presented in Figure 8. In the proposed algorithm, at first, the feasibility of the given storage capacity is evaluated. If the given storage capacity is feasible, a location-specific MILP model is formulated considering the harvest quantity of the respective crop breeds or population to be harvested. The objective is to finish the harvesting within the minimum harvesting week considering the storage capacity. In a situation where all crop breeds can be harvested with the given storage capacity, the model is considered feasible, and the output is regarded as an optimal harvesting schedule leading to an optimal planting schedule.

As indicated in Figure 8, when the feasibility check is not passed, the algorithm will then implement a three-step optimization model. In the first step, the capacity model, the objective is to identify the minimum storage capacity for which all crop breeds can be harvested, respecting their planting window. The output of the capacity model is an input for the second step, the peak-week identifier model. In the peak-week identifier model, the upper bound for additional capacity is fixed by the result of the capacity model, and the peak weeks requiring additional capacity are identified. In the final step, the allocation model, capacity constraints are modified for peak weeks. This algorithm aims to minimize the total additional capacity required for harvesting considering planting windows. Finally, the allocation model is solved to have an optimal harvesting schedule wherefrom the optimal planting schedule can be computed. This three-step model focuses on minimizing the harvesting quantities beyond storage capacity when total harvesting quantities may exceed storage capacity. In a setting where fixed-storage capacity is not sufficient for a given harvest quantity and predicted GDUs, respecting the planting window, this model can provide an optimal planting schedule.

In the event that storage capacity is sufficient for total harvesting quantities, the model formulation for optimal planting schedule is described in the next portion. The designed model has two binary decision variables, one objective function and three constraints. The objective function is minimizing the total number of weeks respecting storage capacity, harvest quantity, and planting window.

#### Model Formulation (Storage Capacity Feasible With Respective to Harvest Quantity)

**Decision Variables:**


$$X_{i\,j}=\left\{ \begin{array}{c} 1,\mathrm{if}\,\mathrm{population}\,i\,\mathrm{is}\,\mathrm{harvested}\,\mathrm{in}\,\mathrm{week}\,j\\ 0,\text{else} \end{array}\right.$$

$$Y_{j}=\left\{ \begin{array}{c} 1,\mathrm{if}\,\mathrm{any}\,\mathrm{harvesting}\,\mathrm{done}\,\mathrm{on}\,\mathrm{week}\,j\\ 0,\text{else} \end{array}\right..$$

**Objective Function:**


(1) $$m\,i\,n:\sum_{j=1}^{70}Y_{j}$$

**Subjected to:**


(2) ∑i=1NQic⁢Xi⁢j≤Cs⁢Yj*,∀j∈{1, 2, 3,…⁢…⁢70}

(3) $$\sum_{j=1}^{70}X_{i\,j}=1,\forall i\in \left\{1,2,3,\ldots \,\ldots \,N\right\}$$

(4) $$X_{i\,j}\leq a_{i\,j}$$

$$X_{i\,j},Y_{j}\geq 0$$

The capacity constraint indicated by Eq. 2 ensures that the total harvesting quantity is less than or equal to storage capacity for any particular week. The decision variable, *Y_j_* presented in the right-hand side of the equation can have value 0 or 1. When the model assigns “0” to *Y_j_*, it means harvesting will not be done in that *j*th week. This confirms that the model will assign harvesting quantities only to *jth* weeks for which *Y_j_* has a value of one. Since, the model objective is minimizing total harvesting weeks, the harvesting weeks will be selected accordingly. Allocation constraint, given by Eq. 3, ensures that each crop breed is harvested only once in a season. The planting window constraint, given by Eq. 4, ensures that the harvesting week respects the planting window. The decision variable *X*_*ij*_ will be zero for harvesting weeks that corresponds to planting dates beyond the planting window. If no feasible solution is identified, the three-step model formulation with modified capacity constraints will be used to have an optimal planting schedule. The detailed model formulation of each step is described in the following segment.

#### Model Formulation (Storage Capacity Infeasible With Respective to Harvest Quantity)

##### Step-1: Capacity Model

**Decision Variables:**


$$X_{i\,j}=\left\{ \begin{array}{c} 1,\mathrm{if}\,\mathrm{population}\,i\,\mathrm{is}\,\mathrm{harvested}\,\mathrm{in}\,\mathrm{week}\,j\\ 0,\mathrm{else} \end{array}\right.$$

Z=Additional⁢required⁢capacity⁢of⁢the⁢site.

**Objective Function:**


(5) m⁢i⁢n:Z

**Subjected to:**


(6) ∑i=1NQic⁢Xi⁢j≤Cs+Z,∀j∈{1, 2, 3,…⁢…⁢70}

(7) $$\sum_{j=1}^{70}X_{i\,j}=1,\forall i\in \left\{1,2,3,\ldots \,\ldots \,N\right\}$$

(8) $$X_{i\,j}\leq a_{i\,j}$$

$$X_{i\,j},Z\geq 0$$

The objective of the capacity model is to identify minimum additional storage capacity to complete total harvesting. For this, the objective function given by Eq. 1 is updated as Eq. 5, where the objective is to minimize required additional storage capacity. The capacity constraint is modified to Eq. 6 from Eq. 2 by adding Z to *C*_*s*_to allow additional capacity. The Eqs 7, 8 are same as Eqs 3, 4 respectively. The optimal solution found in this model is used as an upper bound for the decision variable of the peak-week identifier model.

##### Step-2: Peak-Week Identifier Model

**Decision Variables:**


$$X_{i\,j}=\left\{ \begin{array}{c} 1,\mathrm{if}\,\mathrm{population}\,i\,\mathrm{is}\,\mathrm{harvested}\,\mathrm{in}\,\mathrm{week}\,j\\ 0,\text{else} \end{array}\right.$$

$$m_{j=}\,\mathrm{Additional}\,\mathrm{required}\,\mathrm{capacity}\,\mathrm{of}\,\mathrm{week}\,j$$

**Objective Function:**


(9) $$m\,i\,n:\sum_{j=1}^{70}m_{j}$$

**Subjected to:**


(10) ∑i=1NQic⁢Xi⁢j≤Cs+mj,∀j∈{1, 2, 3,…⁢…⁢70}

(11) $$\sum_{j=1}^{70}X_{i\,j}=1,\forall i\in \left\{1,2,3,\ldots \,\ldots \,N\right\}$$

(12) $$X_{i\,j}\leq a_{i\,j}$$

(13) $$m_{j}\leq Z$$

$$X_{i\,j},m_{j}\geq 0$$

The new decision variable for the peak-week identifier model is *m*_*j*_,which corresponds to the required additional capacity for week *j*. The objective function of the second step given by Eq. 9 is to minimize the sum of *m_j_*. The output of this model is used as an input for the allocation model. In the allocation model, new capacity *C_s_+m_j_* is used for the weeks requiring additional capacity. This adjustment allows the model to allocate extra harvest quantity to weeks for which *m_j_* is greater than zero and only up to *C_s_+m_j_*. This modification determines the required minimum additional storage capacity for peak weeks; moreover, it ensures entire harvesting is completed utilizing a minimum number of weeks. The allocation constraint and planting window constraint given by Eqs 11, 12 are same as Eqs 3, 4, respectively, and Eq. 13 ensures that additional weekly capacity does not exceed the minimum additional capacity determined in step-1.

##### Step-3: Allocation Model

**Decision Variables:**


$$X_{i\,j}=\left\{ \begin{array}{c} 1,\mathrm{if}\,\mathrm{population}\,i\,\mathrm{is}\,\mathrm{harvested}\,\mathrm{in}\,\mathrm{week}\,j\\ 0,\text{else} \end{array}\right.$$

$$Y_{j}=\left\{ \begin{array}{c} 1,\mathrm{if}\,\mathrm{harvesting}\,\mathrm{done}\,\mathrm{on}\,\mathrm{week}\,j\\ 0,\text{else} \end{array}\right.$$

**Objective Function:**


(14) $$m\,i\,n:\sum_{j=1}^{70}Y_{j}$$

**Subjected to:**


(15) ∑i=1NQic⁢Xi⁢j≤(Cs+mj)*Yj,∀j∈{1, 2, 3,…⁢…⁢70}

(16) $$\sum_{j=1}^{70}X_{i\,j}=1,\forall i\in \left\{1,2,3,\ldots \,\ldots \,N\right\}$$

(17) $$X_{i\,j}\leq a_{i\,j}$$

$$X_{i\,j},Y_{j}\geq 0$$

In the third step, the allocation model, the objective function is similar to Eq. 1, minimizing the total number of harvesting weeks. The capacity constraint in Eq. 15 is almost identical to capacity constraint in Eq. 2 with only difference of *m_j_* added to *C_s_*. This modification allows the model to allocate some additional harvesting quantities in peak week. The purpose of this three-step method is to identify the peak weeks when harvested quantities are exceeding the storage capacity. Identification of those weeks will help the decision-maker for timely management actions. The decision-maker may decide to rent extra capacities for those weeks or utilize a third-party storage facility.

#### Case-2: Flexible Storage Capacity

**FIGURE 9:**
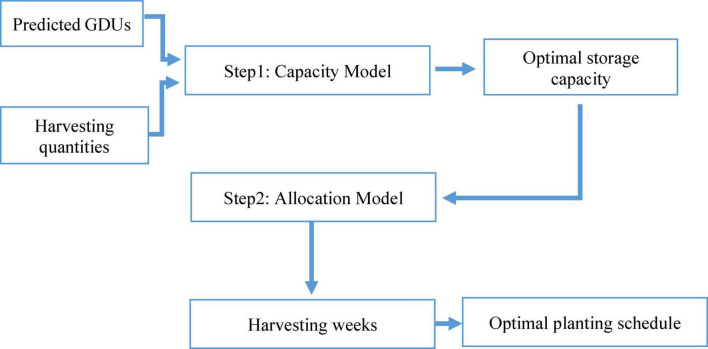
Proposed algorithm for optimal planting schedule for flexible storage capacity.

In case-1, we assume the storage capacity of a site is known or already built. However, to decide on the storage capacity of a new location the preceding model is not applicable. Because, case-1 do not provide information about required storage capacity. Thus, we considered case-2, when the required minimum storage capacity has to be identified. Case-2 can be considered as an extension to case-1, the proposed algorithm for case-2 is illustrated in Figure 9, consisting of two steps. The first step, capacity model focuses on identifying the minimum storage capacity for harvesting, respecting the planting window. The output of the capacity model, the optimal storage capacity is the input for the second step, the allocation model, where the harvesting week for each crop breed is proposed. The second step of case-2 is similar to case-1 in terms for objective function and constraints.

Another approach to solving the model without this two-step method is quadratic programming, simultaneously optimizing storage capacity and total harvesting week. However, the proposed algorithm considers that storage capacity will be determined way before harvesting or planting and will not be updated simultaneously with planting. Both storage capacity and harvesting week do not vary side by side; thus, site optimal storage capacity is identified before finding the optimal planting schedule.

#### Model Formulation (Flexible Storage Capacity)

##### Step -1: Capacity Model

**Decision Variables:**


$$X_{i\,j}=\left\{ \begin{array}{c} 1,\mathrm{if}\,\mathrm{population}\,i\,\mathrm{is}\,\mathrm{harvested}\,\mathrm{in}\,\mathrm{week}\,j\\ 0,\mathrm{else} \end{array}\right.$$

$$C_{z}=\mathrm{Site}\,\mathrm{capacity}.$$

**Objective Function:**


(18) $$m\,i\,n:C_{z}$$

**Subjected to:**


(19) ∑i=1NQic⁢Xi⁢j≤Cz,∀j∈{1, 2, 3,…⁢…⁢70}

(20) $$\sum_{j=1}^{70}X_{i\,j}=1,\forall i\in \left\{1,2,3,\ldots \,\ldots \,N\right\}$$

(21) $$X_{i\,j}\leq a_{i\,j}$$

$$X_{i\,j},C_{z}\geq 0$$

The model formulation for the capacity model is given by Eqs 18–21, where the objective is to determine minimum storage capacity. The objective function given by Eq. 18 focus on identifying minimum storage capacity that will be sufficient for harvesting. The requirement of a minimum number of harvesting weeks is not considered in this step. Thus, *Y_j_* is not a decision variable in this step and not included in capacity constraint in Eq. 19. The allocation constraints and planting window constraints given by Eqs 20, 21 are similar to Eqs 3, 4, respectively.

##### Step -2: Allocation Model

**Decision Variables:**


$$X_{i\,j}=\left\{ \begin{array}{c} 1,\mathrm{if}\,\mathrm{population}\,i\,\mathrm{is}\,\mathrm{harvested}\,\mathrm{in}\,\mathrm{week}\,j\\ 0,\text{else} \end{array}\right.$$

$$Y_{j}=\left\{ \begin{array}{c} 1,\mathrm{if}\,\mathrm{harvesting}\,\mathrm{done}\,\mathrm{on}\,\mathrm{week}\,j\\ 0,\text{else} \end{array}\right.$$

**Objective Function:**


(22) $$m\,i\,n:\sum_{j=1}^{70}Y_{j}$$

**Subjected to:**


(23) ∑i=1NQic⁢Xi⁢j≤Cz*Yj,∀j∈{1, 2, 3,…⁢…⁢70}

(24) $$\sum_{j=1}^{70}X_{i\,j}=1,\forall i\in \left\{1,2,3,\ldots \,\ldots \,N\right\}$$

(25) $$X_{i\,j}\leq a_{i\,j}$$

$$X_{i\,j},Y_{j}\geq 0$$

Using the optimal storage capacity from the capacity model, in second step, the allocation model, a harvesting schedule is developed by implementing Eqs 22–25 to have a minimum harvesting week respecting storage capacity, which are similar to Eqs 1–4. The difference between these equations are *C_z_*. In Eq. 2 *C_z_* is an input to the model, whereas, in Eq. 23 *C_z_* is calculated from earlier step, the capacity model. From this optimal harvesting schedule, we can calculate the optimal planting date for different crop breeds.

For both fixed and flexible storage capacity, one decision variable is *X*_*ij*,_ giving the information of which crop breed to be harvested on which week. Once the optimal harvesting week is identified, corresponding planting date can be determined using the matrix *H*_*id*_, calculated earlier to create, *a*_*ij*_. The parameter *H*_*id*_, is the harvesting week for plant *i*, if planting is done on day *d.* From this, the planting date corresponding to the optimal harvesting week is calculated. This proposed method can provide optimal planting schedules for both fixed-site capacities and propose the optimal storage capacity for a new location.

## Case Study

As stated, this study is motivated by the logistic challenges faced in the commercial corn production process with a focus on the planting and harvesting stages. Once the corn reaches the required GDUs, harvesting has to be done within a week. There are two scenarios, one with fixed storage capacity and the other with flexible storage capacity. For fixed storage capacity, the objective is to perform planting so that the weekly harvesting quantity is within storage capacity and the heat requirements in the planting stage are also satisfied. It can be attained by an optimal planting schedule that respects both planting and harvesting constraints. For flexible storage capacity, minimum optimal storage capacity has to be determined prior to proposing an optimal planting schedule.

To demonstrate and validate the proposed model, a case study based on Syngenta Crop Challenge (2021) has been conducted, which includes two different geographical locations with two yield scenarios (Syngenta Crop Challenge, 2021 - the challenge). Apart from the historical GDUs, no additional geographical information is available for those locations. For this reason, we used the GDUs prediction model discussed in section “Growing Degree Units Prediction” to predict the GDUs for the upcoming season. This research incorporates the concept of utilizing GDUs to identify harvesting dates proposed by Zyskowski and Jamieson (2014). Apart from calculating accumulated GDUs, experimental works showed that, on average, corn becomes ready for harvesting within 95–110 days (Schmidt and Hallauer, 1966; Cox et al., 1994). Another interesting finding is that when corns are planted at a lower temperature, they tend to reach maturity with a low heat requirement (Baum et al., 2019; Nielsen, 2021). Considering this information, we set an upper bound of 120 days to the number of days needed to achieve the required accumulated GDUs. Then we used the predictions as an input for the planting model introduced in section “Optimization Model for Planting” to design the optimal plating schedule for different locations and harvest quantities. The prediction model is developed in Python 3.6.10, and the plant scheduling model is solved using Gurobi 9.1.0 optimizer (Gurobi, 2018) in the Matlab platform.

### Case-1: Fixed Storage Capacity

**FIGURE 10:**
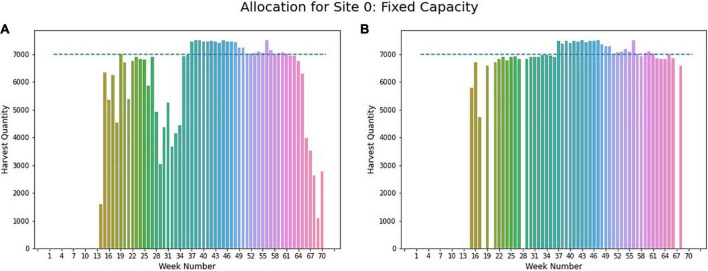
Weekly allocation site 0 (fixed capacity). (A) Initial weekly allocation. (B) Final weekly allocation.

The proposed algorithm in section “Case-1: Fixed Storage Capacity” is implemented for two locations named site 0 and site 1, with a fixed capacity of 7,000 ears and 6,000 ears count of corn, respectively. After the implementation, it is observed that given predicted GDUs for site 0 and planting windows for different crop breeds or populations, there is no feasible solution for site 0 respecting the given capacity. For this reason, the three-step model with a modified capacity constraint is utilized to determine the optimal weekly harvesting and planting schedule. As presented in Figure 10, weekly harvesting quantities exceed the capacity limit of 7,000 ears of corn illustrated with the blue dashed line. In the first step, for site 0, it is found that a minimum storage capacity of 7,500 ears is required to harvest all crop breeds respecting their planting window. This capacity is used in steps 2 and 3 to obtain the optimal harvesting schedule.

Figure 10A provides the weekly harvest quantity obtained from step 2, where it can be seen that week 37 to 49 requires some additional capacity for harvesting. This information is used to find the final optimal planting schedule from step 3, where for weeks 37 to 49, storage capacity is relaxed to 7,500 ear counts of corn to have an optimal solution. The final weekly allocation for site 0 is given in Figure 10B, where total harvesting quantities are evenly distributed throughout the season.

**FIGURE 11:**
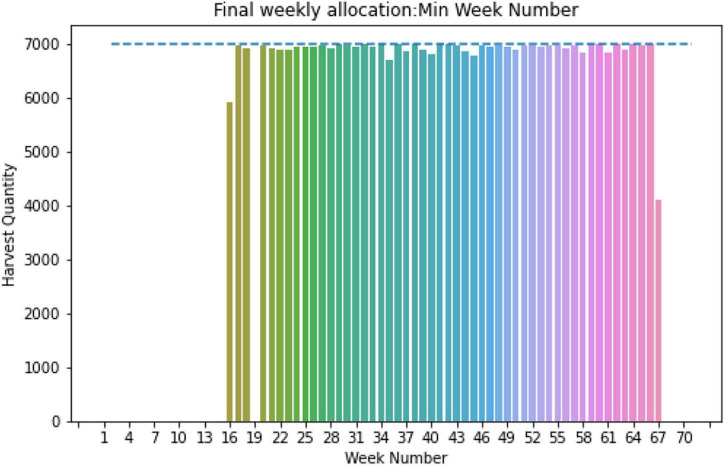
Weekly allocation site 0 (fixed capacity and max 120 days for harvesting).

With further analysis of GDUs of site 0, it was found that the required days for harvesting exceed 120 days for some populations, which is the average maximum required days for harvesting for corn (Schmidt and Hallauer, 1966; Cox et al., 1994). The governing reason for this is, site 0 has a relatively lower temperature for a season. One study conducted in the United States corn belt found that required GDUs reduce on an average of 110 GDUs when planted in early June compared to early May. As in June, there is a drop in temperature that resulted in reduced accumulated GDUs for the maturity of corn (Williams, 2006). Referring to this finding and the temperature trend of site 0, we assumed that for the late planting date, which is mostly in the cooler season, the required GDUs might drop, keeping the days required for harvesting to a limit of 120 days. With this modification, the algorithm is applied to site 0, and a feasible allocation is found within a given storage capacity of 7,000 ears. The total weekly harvest quantity for site 0 is shown in Figure 11, and weekly allocations are within the storage capacity of 7,000 ears, indicated by the blue dashed line.

**FIGURE 12:**
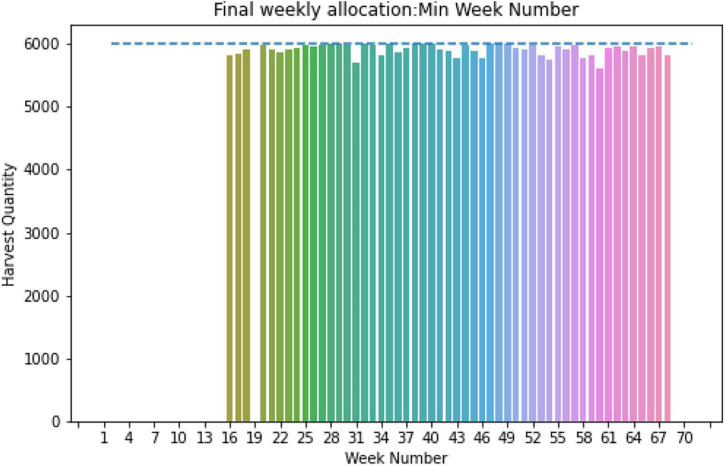
Weekly allocation site 1 (fixed capacity).

Utilizing the same algorithm for site 1, we obtained an optimal strategy for the given storage capacity of 6,000 ears, corresponding to predicted GDUs for site 1, population-wise harvesting quantity, and planting window. For site 1, no additional modification is needed for the required harvesting days as they are with the 120 days limit. The optimal weekly allocation for site 1 is presented in Figure 12, where for all weeks, the total harvested quantity is below 6,000 ears count of corn which is the blue dashed line.

### Case-2: Flexible Storage Capacity

For case-2, flexible storage capacity, for both sites no fixed storage capacity is given. So, the algorithm developed in section “Case-2: Flexible Storage Capacity” is applied at first to determine the minimum optimal capacity for both sites. Implementing the first step of the proposed algorithm, we found a minimum storage capacity of 9,800 ears and 7,900 ears of corn for site 0 and site 1. In the second step of the algorithm, these optimal capacities are used to determine the optimal planting schedule to have a minimum number of total harvesting weeks.

**FIGURE 13:**
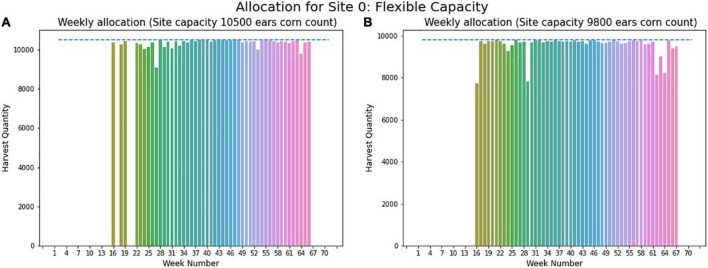
Weekly allocation site 0 (flexible capacity). (A) Weekly allocation (Site capacity 10500 ears corn count). (B) Weekly allocation (Site capacity 9800 ears corn count).

Figure 13A represents the weekly allocation for site 0, without the limit on required harvesting days to be 120 days. In this case, the required storage capacity is 10,500 ears of corn, given by the dashed line in the figure. Whereas, Figure 13B represents the weekly allocation with maximum harvesting days of 120 days, and with this modification, the capacity of site needed reduces to 9,800 ears, presented by the blue dashed line in Figure 13B. The literature advocate for limiting required harvesting days considering site 0 climates. Therefore, the final results were found by restricting the harvesting days necessary to be 120 days.

**FIGURE 14:**
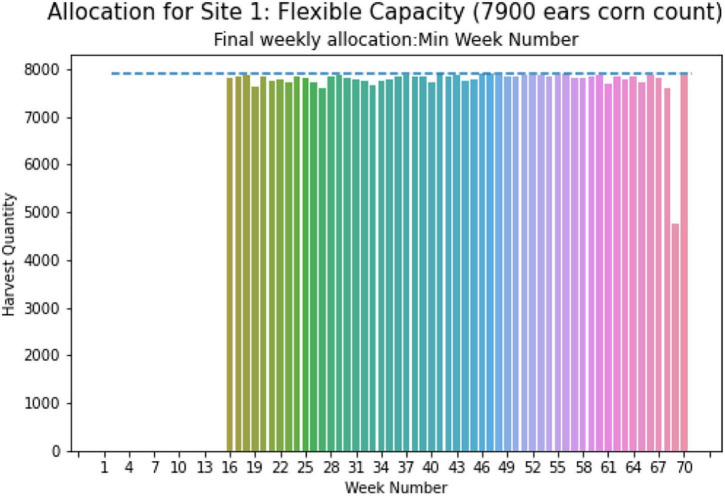
Weekly allocation site 1 (flexible capacity).

Figure 14 illustrates the weekly total harvest quantity for site 1 in case-2 with the minimal optimal capacity of 7,900 ears given by the dashed line. The weeks required for harvesting ranges from week 16 to week 70. From Figure 14, it is evident that the harvest quantities are uniform within this range.

The outcomes of the proposed algorithms for both cases have been listed in Table 4. The first evaluation matrix is “Maximum Absolute Deviation,” which provides information on the maximum difference between storage capacity and weekly allocated quantity. It should be noted that a lower value is preferable, indicating proper utilization of storage capacity since weekly allocated quantities are closer to storage capacity. The second evaluation matrix is “Median Absolute Deviation”, which gives an idea about the overall deviation from harvest quantity and available capacity. These matrixes evaluate the performance of the scheduling model for planting. A lower value indicates that the model is capable of proving an optimal planting schedule respecting storage capacity. The third evaluation matrix is “Total allocation,” the total number of weeks of a season used for harvesting. For this case study, the entire season is of 70 weeks; thus, it is expected to complete the harvesting within fewer weeks.

**TABLE 4:** Evaluation matrix.

Evaluation matrix	Site 0		Site 1	
	Fixed site capacity (Case-1)	Flexible site capacity (Case-2)	Fixed site capacity (Case-1)	Flexible site capacity (Case-2)
Maximum absolute deviation	2,883	2,057	394	3,148
Median absolute deviation	16.5	49.5	29.5	48.5
Total allocation (week number)	51	52	52	55
Proposed capacity (ears of corn)	N/A	9,800	N/A	7,900

For site 0 in case-1, in week 67, the weekly allocation is significantly smaller than the storage capacity since by that time, most of the corn breeds or populations have been harvested, and there is not much left for harvesting. For this reason, week 67 has the maximum absolute deviation for site 0 in case-1. In case-2, the maximum absolute deviation from site 0 capacity is about 2,000 ears count of corn resulting from week 62 when harvesting for most of the plant is completed. Though the maximum absolute deviation from storage capacity for site 0 in both cases has a higher value, the smaller value of median of absolute deviation indicates consistent harvested quantities. The total harvesting required 50 weeks and 48 weeks in case 1 and 2, respectively, implying that harvesting will be done engaging fewer weeks. For site 1 in case-1, the maximum absolute deviation is moderate. In contrast, in case-2, there is a higher maximum absolute deviation of around 3,000 ears count of corn occurring in week 69 when not many plants are left to harvest. In both cases for site 1, the median of absolute deviation from storage capacity implies uniform harvested quantities through the season. Similar to site 0, in site 1, total harvesting is performed for cases 1 and 2, requiring fewer weeks, 52 and 55 weeks, respectively.

The four evaluation matrices summarized in Table 4 show that only maximum absolute deviations have a relatively higher value. The weeks corresponding to those maximum absolute deviations are at the beginning of the season or the end. When either the crops are not ready for harvesting, or harvesting is done for most of them, which decode the higher maximum absolute deviations in those timeframes. To summarize, the results show that the proposed model performs well for different yield quantities and locations, providing an optimal planting schedule.

## Conclusion

The research objective of this study is to achieve evenly distributed weekly harvested quantities considering the planting windows and storage capacity. The goal is to reduce food wastage and address the logistical challenges in the harvesting and storage phases. In this study, we propose a two-stage crop planting model that recommends the planting dates considering plant growth as well as logistical and capacity limitations. To accurately predict the maturity and thus harvesting dates, heat requirements and required GDUs need to be followed. An optimization model is then implemented to determine optimal planting dates so that weekly harvesting quantities will not exceed the storage capacity.

Our proposed methodology is divided into two stages: predicting GDUs based on historical data and developing an optimal planting schedule. In the GDUs prediction stage, the selection of the prediction model is essential. The prediction model selected is 1D-CNN as it has a low RRMSE of 7 and 9% for site 0 and site 1, respectively. These predicted GDUs are one of the inputs for the optimization model to determine harvesting dates. The optimization model is designed to complete harvesting with a minimum number of weeks and a consistent weekly harvesting quantity within the bounds of storage capacity. The two-stage decision making model is illustrated for two scenarios: (1) fixed storage capacity and (2) flexible storage capacity. The first stage, the GDUs prediction model, remain the same for both scenarios. However, there are differences in the decision framework design in the second stage, the optimization model stage. Under the scenario of the fixed storage capacity, the second stage is designed to check the adequacy of storage capacity for total harvested quantities. If there is insufficient storage capacity, the optimization model in this stage can identify peak weeks where harvested quantities may exceed the storage capacity, allowing decision-makers to act accordingly. Whereas, for the scenario of the flexible storage capacity, the second stage is constructed to determine both optimal storage capacity and provide consistent weekly harvesting quantities simultaneously. The results from both scenarios demonstrate that weekly harvesting quantities obtained by implementing the planting schedule from the proposed decision making framework are evenly distributed throughout the harvesting phase using fewer harvesting weeks. This validates that the proposed method is robust for varying assumptions and conditions.

This study is subject to a few limitations which suggest future research directions. Firstly, for the GDUs prediction, incorporating soil information may provide better accuracy. Considering additional soil information will help understand the crop growth stages accurately and thus better predict the required days for harvesting. Secondly, the proposed planting decision-making model can be improved to be adaptive to the real-life situation by including additional factors such as fertilization and irrigation. Lastly, designing a user interface for broader dissemination will make it accessible to farmer and agriculture practitioners. These can be reserved for future research directions.

## Data Availability Statement

The original contributions presented in the study are included in the article/supplementary material, further inquiries can be directed to the corresponding author.

## Author Contributions

SS conducted the research and wrote the first draft of the manuscript. GH supervised the research, provided guidance, and reviewed and edited the manuscript. Both authors contributed to the article and approved the submitted version.

## Conflict of Interest

The authors declare that the research was conducted in the absence of any commercial or financial relationships that could be construed as a potential conflict of interest.

## Publisher’s Note

All claims expressed in this article are solely those of the authors and do not necessarily represent those of their affiliated organizations, or those of the publisher, the editors and the reviewers. Any product that may be evaluated in this article, or claim that may be made by its manufacturer, is not guaranteed or endorsed by the publisher.
